# Recurrent Stent Migration in a Patient With a Gastropleural Fistula Following a Single Anastomosis Sleeve Ileal Bypass Procedure Resulted in Bowel Resection

**DOI:** 10.7759/cureus.94877

**Published:** 2025-10-18

**Authors:** Amal Alqarafi, Taghreed Albalawi, Fahad Bamehriz

**Affiliations:** 1 General Surgery Division, Department of Surgery, King Fahad Hospital, Madinah, SAU; 2 Minimally Invasive and Bariatric Surgery, Upper Gastrointestinal Surgery Unit, Department of Surgery, King Fahad Hospital, Madinah, SAU; 3 General Surgery, College of Medicine, King Khalid University Hospital, King Saud University, Riyadh, SAU

**Keywords:** bariatric surgery, endoscopic stent, gastroplural fistula, single anastomosis sleeve ileal bypass, stent migration

## Abstract

Gastropleural fistula (GPF) is a rare complication following bariatric surgery, most commonly reported after laparoscopic sleeve gastrectomy, with no previously documented cases following single anastomosis sleeve ileal bypass (SASI). Endoscopic stenting is considered the primary management strategy for post-bariatric gastric fistulas and generally demonstrates a high success rate. However, stent migration remains a significant complication that can result in considerable morbidity.

We report a case of a 32-year-old male who developed a chronic GPF following the SASI procedure. Initial management involved endoscopic stenting; however, the patient experienced recurrent stent migration, which ultimately resulted in small bowel obstruction. This complication necessitated an exploratory laparotomy and subsequent bowel resection. The persistent stent migration observed in this case may be linked to abnormal gastrointestinal motility changes following bariatric surgery.

This case highlights the need to consider early surgical intervention in select cases and emphasizes the importance of prospective studies investigating post-SASI motility changes.

## Introduction

Gastropleural fistula (GPF) is a rare and life-threatening complication following bariatric surgery, most often resulting from stapler line disturbance and typically presenting with non-specific clinical symptoms [1].

Endoscopic therapy, particularly stenting, has become the initial and primary modality of treatment for gastric leaks and fistulas after bariatric surgery, with success rates reaching up to 92%, especially as stent designs have evolved to suit post-bariatric cases [2]. Despite these advancements, stent migration remains the most common complication occurring in 23% of cases, as reported by a recent systematic review and meta-analysis [3].

In light of this, we present a patient who developed a GPF after a single anastomosis sleeve ileal bypass (SASI). Notably, the patient failed endoscopic therapy and experienced recurrent stent migration, with the last migration leading to bowel obstruction and bowel ischemic changes, necessitating exploratory laparotomy and subsequent bowel resection. The failure of multiple endoscopic stent interventions emphasizes that early surgical intervention could be a suitable first-line option for leaks or fistulas after SASI.

## Case presentation

**Figure 1:**
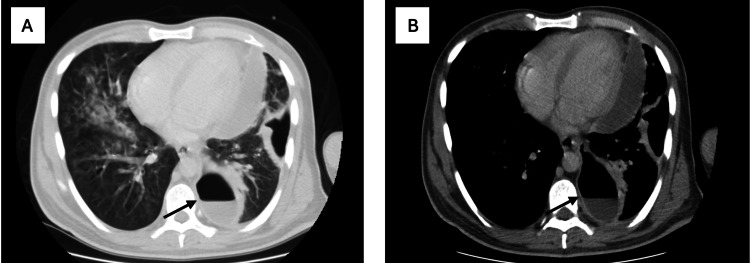
(A) Chest CT scan (lung window) showing bilateral parenchymal infiltrations suggestive of pneumonia. (B) Chest CT scan (mediastinal window) showing a left-sided cavitation communicating between the gastroesophageal junction (GEJ) and the left lung, suggestive of a gastropleural fistula (GPF).

The patient was a 32-year-old male who underwent the SASI procedure for obesity. Eight months after the surgery, the patient presented to the emergency department (ED) with a productive cough, fever, abdominal pain, and hypoxia. Contrast CT scan of the chest showed left-sided pneumonia with cavitation suggestive of GPF (Figure 1), as well as inflammatory changes around the gastroesophageal junction (GEJ) consistent with leak.

The patient was admitted to the intensive care unit (ICU) and intubated due to severe respiratory distress, started on empirical IV antibiotics, antifungal, and total parenteral nutrition (TPN). The next day, the patient underwent left video-assisted thoracoscopic surgery (VATS), empyema was drained, and a chest tube was inserted.

After stabilization of the patient's condition, esophagogastroduodenoscopy (EGD) with fluoroscopy was done, and showed a fistula opening 4 cm above the GEJ. A covered metallic esophageal stent measuring 28 mm x 280 mm was inserted and fixed with two metallic clips, one 3 cm proximal to the fistula opening and the other distally at the duodenal opening. A follow-up imaging done the next day showed migration of the stent to the colon. A colonoscopy was performed, and the stent was successfully removed. Then, another Mega covered stent (24 mm x 230 mm) was inserted and fixed with two metallic clips. Follow-up imaging was performed a few hours later, and the stent migrated again to the colon and passed spontaneously. A nasojejunal tube (NJT) was inserted under fluoroscopic guidance to initiate feeding. Four weeks later, a follow-up EGD was done, which showed no fistula opening. NJT was removed, and oral intake was started.

Three months later, a follow-up CT scan was done and showed a perigastric collection with fat stranding extended up to the left lung, consistent with chronic gastropulmonary fistula. A double pigtail stent was inserted to drain the collection.

After eight weeks of follow-up, EGD and imaging revealed non-resolution of the GPF. The patient was prepared for laparoscopic exploration with GPF closure. Intraoperative findings revealed two fistula tracts, a GPF and a gastrobronchial fistula. Resection of the fistula tract was done using Endo GIA (Medtronic, Dublin, Ireland), and oversewing of the distal esophagus was done. The patient was febrile postoperatively, and a contrast CT scan of the abdomen showed a leak at the distal esophagus. EGD was performed, and a metallic esophageal stent (24 mm x 230 mm) was applied and secured with hemoclips. Unfortunately, it was migrated, and then another covered metallic esophageal stent of 36 mm x 230 mm was applied.

**Figure 2:**
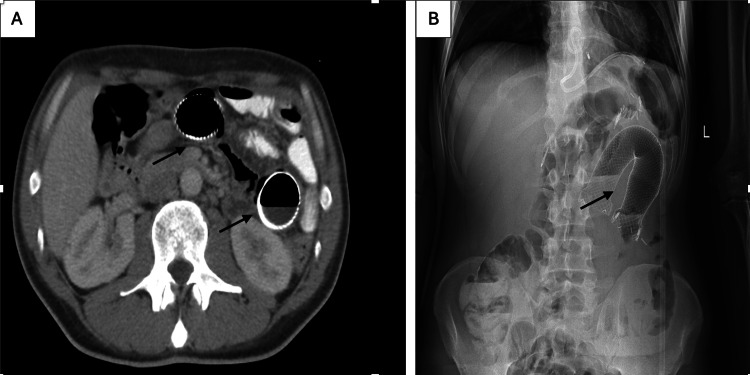
(A) Erect abdominal X-ray showing a self-expandable metal stent (SEMS) in the left upper quadrant of the abdomen. (B) Abdominal CT scan showing a migrated 36 mm SEMS in the proximal jejunum, associated with bowel dilation.

**Figure 3:**
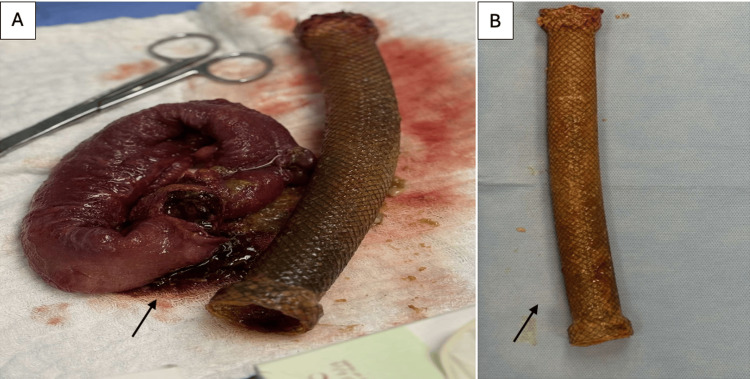
(A) An operative picture demonstrating the resected ischemic bowel segment. (B) Image demonstrating the length and size of the stent.

Two weeks later, the patient presented to the ED with abdominal pain and vomiting. A CT scan of the abdomen showed a migrated stent in the proximal jejunum. Due to worsening of symptoms, the patient was taken for an exploratory laparotomy. The migrated stent was in the proximal jejunum, causing obstruction with bowel ischemic changes. Resection with primary anastomosis was done (Figures 2, 3).

## Discussion

SASI is a bariatric surgical procedure modified by Mahdy et al. in 2016 from the Santoro procedure. It involves a simple loop connecting the gastric antrum to the ileum. The technique combines volume restriction with preserved food transit through the gastrointestinal system while avoiding the side effects of malabsorptive surgery. Compared to other bariatric procedures, SASI achieves similar improvements in obesity-related comorbidities, such as diabetes mellitus (DM) and hypertension (HTN), and a similar reduction in total weight loss [4-6].

GPF is one of the feared complications after bariatric surgery. It is defined as an abnormal communication between the stomach and the pleural space. Markowttz and Herter first described this condition in 1960 [7]. GPF after bariatric procedures is rare, with an incidence of 0.2%-0.37%. A higher percentage is noted following sleeve gastrectomy compared to Roux-en-Y gastric bypass (RYGB) [7]. Several reports exist on GPF after sleeve gastrectomy and RYGB. However, no prior cases have been reported following SASI. This makes our case a unique contribution and expands current understanding of SASI-related complications. The clinical presentation can be vague, and imaging is essential to make a diagnosis. Despite recent updates, no clear protocol or guideline exists for management. Published systematic reviews recommend initiating treatment with noninvasive measures and reserving surgical intervention as a last resort in stable patients.

Endoscopic intervention has become the cornerstone in managing post-bariatric gastric fistulas, with various modalities utilized, including fibrin glue application, stenting, clipping, and endosuturing. Among these, the use of stents can improve and simplify management by sealing leaks immediately and facilitating early enteral intake [8]. There are three main types of self-expanding metal stents (SEMS): fully covered, partially covered, and uncovered; however, fully covered and partially covered SEMS are used more frequently as their outer covering can help seal a leak or perforation [9]. While stents demonstrate excellent efficacy, a key limitation is migration, which in some instances may lead to secondary injuries such as perforation or obstruction and require subsequent interventions. To address this, multiple techniques have been developed to minimize stent migration. Over-the-scope clips and sutures, the most popular and commonly deployed methods, significantly lower migration rates, which typically range from 15% to 40%. In support of these efforts, a meta-analysis published in 2025 identified several risk factors for stent migration, including stents with a diameter of less than 20 mm, a length of less than 100 mm, a plastic composition, and a fully covered design. Conversely, stents using antimigration technology show lower migration rates [10].

In this report, the patient had a history of a SASI procedure and subsequently developed a chronic GPF. The fistula did not respond to multiple attempts of endoscopic management. Specifically, several stents were placed at different times; some migrated within hours, while others migrated after several days, despite being Mega stents (diameter > 20 mm and length > 100 mm) with anti-migration techniques, such as metallic clips. The last stent led to a complication of bowel obstruction with ischemic changes, which required exploratory laparotomy and small bowel resection. Abnormal gastrointestinal motility has been reported following sleeve gastrectomy and RYGB, and similar alterations may occur after SASI. These motility changes could explain the unusually high rate of stent migration observed in our patient, despite using mega stents with anti-migration techniques. However, the exact mechanism remains poorly understood and requires further investigation [11].

The failure of multiple endoscopic stent interventions in this case emphasizes that endoscopic therapy may not always be a suitable first-line option for leaks/fistulas after SASI. Alternative strategies, including early surgical intervention or hybrid approaches, should be considered in selected patients. Given the rising popularity of SASI, there is an urgent need for multicenter registries to capture rare complications as well as prospective studies focusing on motility changes post SASI and comparative studies to evaluate management outcomes of leaks/fistulas across different bariatric procedures.

## Conclusions

In summary, this case highlights a rare but severe complication of SASI, demonstrating the limitations of current endoscopic management. It emphasizes the importance of comprehensive clinical data, well-designed studies, and standardized protocols to optimize management and enhance patient outcomes in the context of this evolving bariatric procedure.

## References

[1] Koussayer B, Kattih M, Nester M, Peterson P, DuCoin CG (2023). Gastropleural fistula presenting as a complication of gastric sleeve surgery: a case report. Cureus.

[2] Billmann F, Pfeiffer A, Sauer P (2022). Endoscopic stent placement can successfully treat gastric leak following laparoscopic sleeve gastrectomy if and only if an esophagoduodenal megastent is used. Obes Surg.

[3] Rogalski P, Swidnicka-Siergiejko A, Wasielica-Berger J (2021). Endoscopic management of leaks and fistulas after bariatric surgery: a systematic review and meta-analysis. Surg Endosc.

[4] Tarnowski W, Barski K, Jaworski P, Binda A, Kudlicka E, Wąsowski M, Jankowski P (2022). Single anastomosis sleeve ileal bypass (SASI): a single-center initial report. Videosurgery.

[5] Mahdy T, Emile SH, Madyan A (2020). Evaluation of the efficacy of single anastomosis sleeve ileal (SASI) bypass for patients with morbid obesity: a multicenter study. Obes Surg.

[6] Emile SH, Mahdy T, Schou C, Kramer M, Shikora S (2021). Systematic review of the outcome of single-anastomosis sleeve ileal (SASI) bypass in treatment of morbid obesity with proportion meta-analysis of improvement in diabetes mellitus. Int J Surg.

[7] Markowttz AM, Herter FP (1960). Gastro-pleural fistula as a complication of esophageal hiatal hernia. Ann Surg.

[8] Parkash O, Sohail Z, Khalid N (2023). Endoscopic stent placement for the management of gastro-pleural and gastro-cutaneous fistula post laparoscopic sleeve gastrectomy: a case report. J Med Case Rep.

[9] Law R, Prabhu A, Fujii-Lau L, Shannon C, Singh S (2018). Stent migration following endoscopic suture fixation of esophageal self-expandable metal stents: a systematic review and meta-analysis. Surg Endosc.

[10] Heutlinger O, Acharya N, Kharabaf S (2025). A systematic review and meta-analysis of factors associated with esophageal stent migration and a comparison of antimigration techniques. J Gastrointest Surg.

[11] Gala K, Ghusn W, Abu Dayyeh BK (2024). Gut motility and hormone changes after bariatric procedures. Curr Opin Endocrinol Diabetes Obes.

